# Primary Testicular Lymphoma Mimicking Germ-Cell Tumor: A Case Report

**DOI:** 10.7759/cureus.48990

**Published:** 2023-11-18

**Authors:** Murtadah Alnemer, Jomana M Felemban, Ali Mansoor, Sohail A Butt

**Affiliations:** 1 Urology, Dammam Medical Complex, Dammam, SAU; 2 Urology, King Fahad Specialist Hospital, Dammam, SAU; 3 Pathology, Dammam Medical Complex, Dammam, SAU

**Keywords:** radical orchiectomy, primary testicular lymphoma, non-hodgkin lymphoma, b-cell, testicular lymphoma

## Abstract

There are 1% to 2% of lymphoma cases that include the testis as primary testicular non-Hodgkin lymphoma (NHL). In 35% of cases, it involves both testes and is usually seen as a painless testicular mass. Therefore, in most cases, the management option is radical orchiectomy. The overall prognosis in these cases is poor, as most cases are associated with systemic disease. We report a case of a 42-year-old male who presented with painless right scrotal swelling for three months. The only serologic marker of solid tumors that was elevated was βHCG; others were unremarkable. Ultrasonography was initially ordered as well and showed a heterogeneous intra-testicular lesion of relatively low echogenicity. According to the given age, epidemiology, and clinical presentation, the suspicion of a germ cell tumor was highly likely. Therefore, a right radical inguinal orchiectomy was done, and the specimen was sent for histopathology, which came back as B-cell non-Hodgkin lymphoma. The clinical presentation and the overall picture of the investigations made in this case mimicked a germ cell tumor presentation.

## Introduction

Primary testicular lymphoma is a subtype of non-Hodgkin lymphoma (NHL) that arises in the testicles and has a high incidence of bilateral involvement of the testicles and metastasis [1,2]. Most testicular lymphomas are diffuse large B-cell types, which is considered the most common cause of testicular mass. Usually, it is present in men over the age of 50 [3-6]. Diagnostically, it can be challenging, as there is a chance to mistake it for other testicular tumors. However, the initial treatment offered will be radical orchiectomy, while staging and subsequent therapy should be done by hematology-oncology. The overall prognosis in these cases is poor [6,7].

## Case presentation

This is the case of a 42-year-old Sudanese male who is not known to have any pertinent medical illnesses. He presented to the urology clinic with a complaint of painless right scrotal swelling for three months. A physical examination revealed a right solid scrotal mass and a normal left testis. The only serologic marker of solid tumors that was elevated was βHCG; others were within the normal range. Imaging studies were done as well; the ultrasound showed an enlarged right testis with a heterogeneous hypoechoic intratesticular lesion. The computed tomography (CT) revealed no metastasis and confirmed an enlarged right testis with hypodense central foci. Then, the patient underwent a right inguinal orchiectomy, and the specimen was sent for histopathology.

Results

Laboratory Tests

The serum alpha-fetoprotein (αFP) and serum lactate dehydrogenase (LDH) levels were normal. Serum beta-human chorionic gonadotropin (βHCG) was elevated, reaching 100 IU/L (0.02-0.8 IU/L).

Radiology

**Figure 1:**
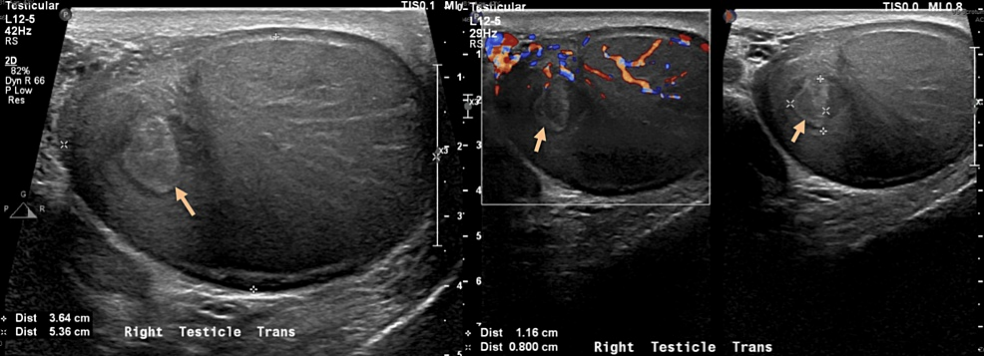
Shows right testicular ultrasonography and doppler ultrasound demonstrating a heterogeneous intratesticular lesion with relatively low echogenicity.

The ultrasonography showed right testicular enlargement and a heterogeneous intratesticular lesion of relatively low echogenicity (Figure 1).

**Figure 2:**
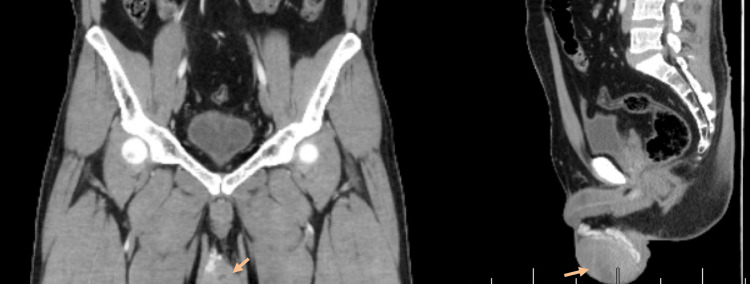
A pelvic CT (coronal and sagittal view) revealing a heterogeneous enlarged right testes with central foci of hypodensity.

**Figure 3:**
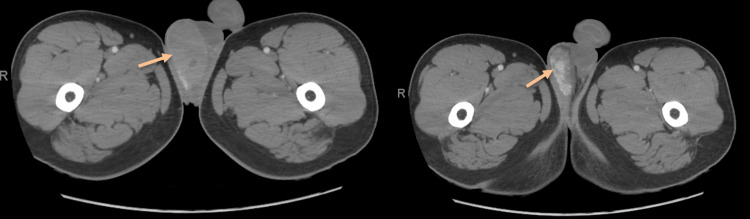
A pelvic CT (transverse view) revealing a heterogeneous enlarged right testes with central foci of hypodensity.

CT was done afterward and revealed heterogeneous right testicular enlargement with central foci of hypodensity suggestive of necrosis, with minimal right hydrocele and no signs of metastasis (Figures 2-3).

Pathologic Features

Gross examination revealed a gray-white, firm mass replacing the entire testicular parenchyma, with a central soft yellow area representing necrosis. The tumor was not extending into the capsule or epididymis. The tunica, epididymis, and spermatic cord appeared unremarkable.

Microscopic Features

**Figure 4:**
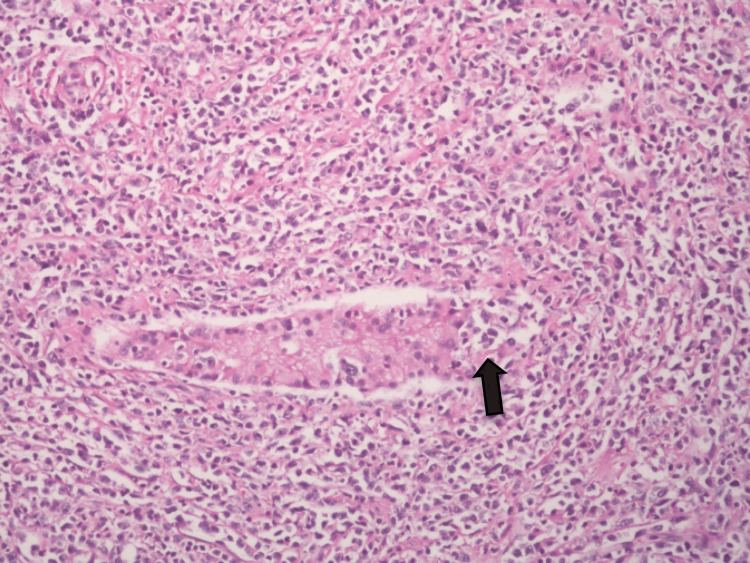
H&E ×100: reveals diffuse infiltration of the testis by atypical lymphoid cells. Some could be seen infiltrating the residual seminiferous tubule.

Effacement of normal testicular architecture and replacement by diffuse lymphoid cell infiltrate was also seen infiltrating the few residual seminiferous tubules (Figure 4).

**Figure 5:**
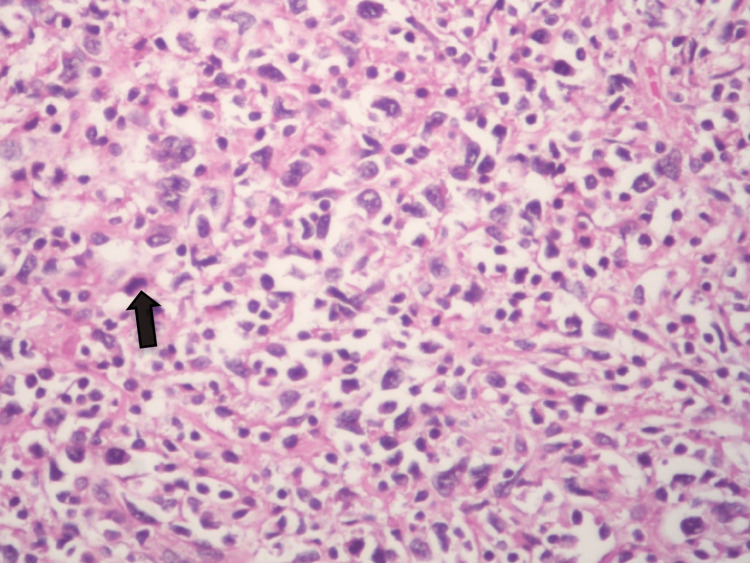
H&E ×400: shows medium to large atypical mitotically active lymphoid cells showing prominent nucleoli.

The lymphoid population comprised medium-to-large lymphoid cells showing round, oval, or pleomorphic nuclei and prominent nucleoli scattered in the background of mature, smaller lymphoid cells and histiocytes (Figure 5). Focal coagulative necrosis was present. Large numbers of lymphoid cells were also present in the spermatic cord vessels and the peri-vascular stroma.

Immunohistochemistry

**Figure 6:**
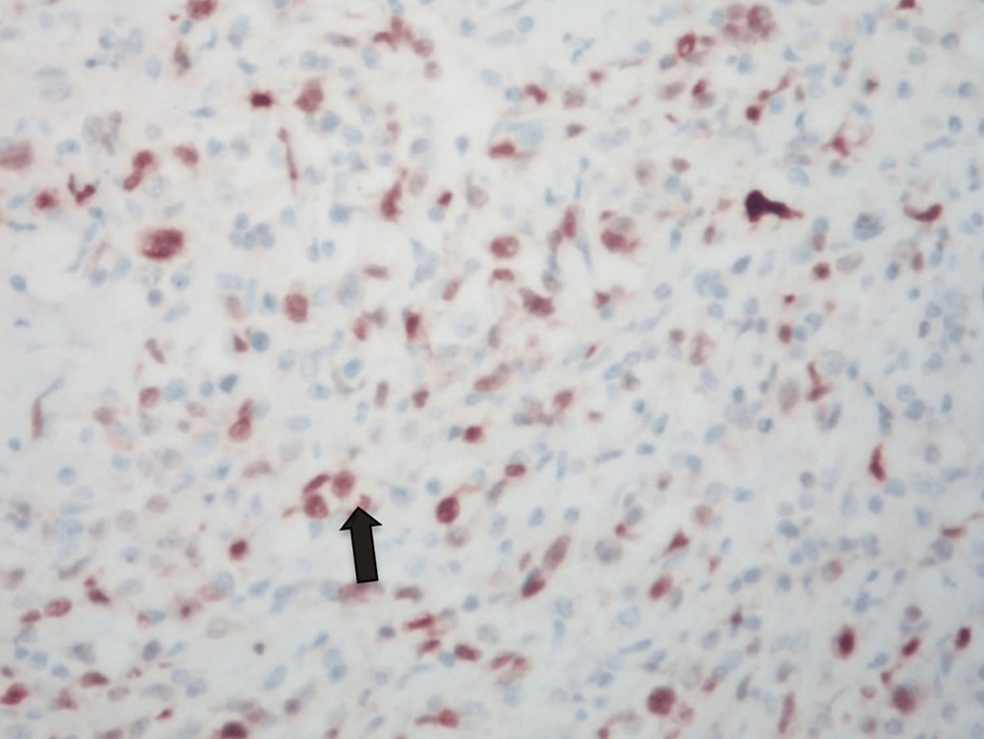
IHC ×400 shows MUM1 expression in large lymphoid cells. MUM1: multiple myeloma oncogene 1.

**Figure 7:**
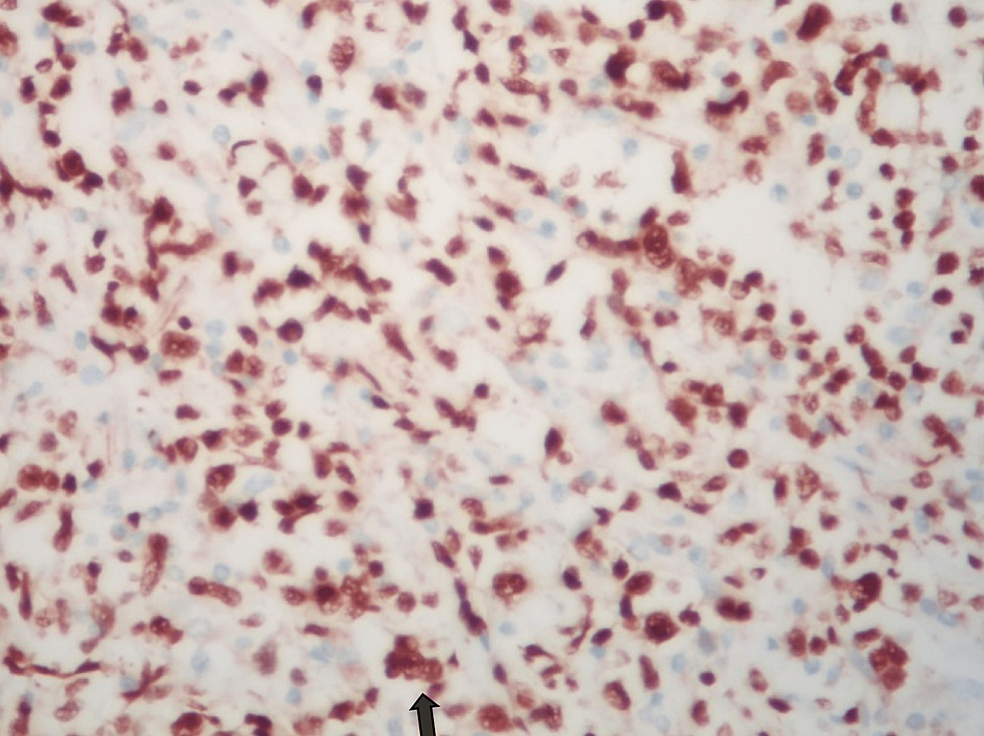
IHC ×400 reveals PAX5 expression. A nuclear marker indicating B cell histogenesis.

**Figure 8:**
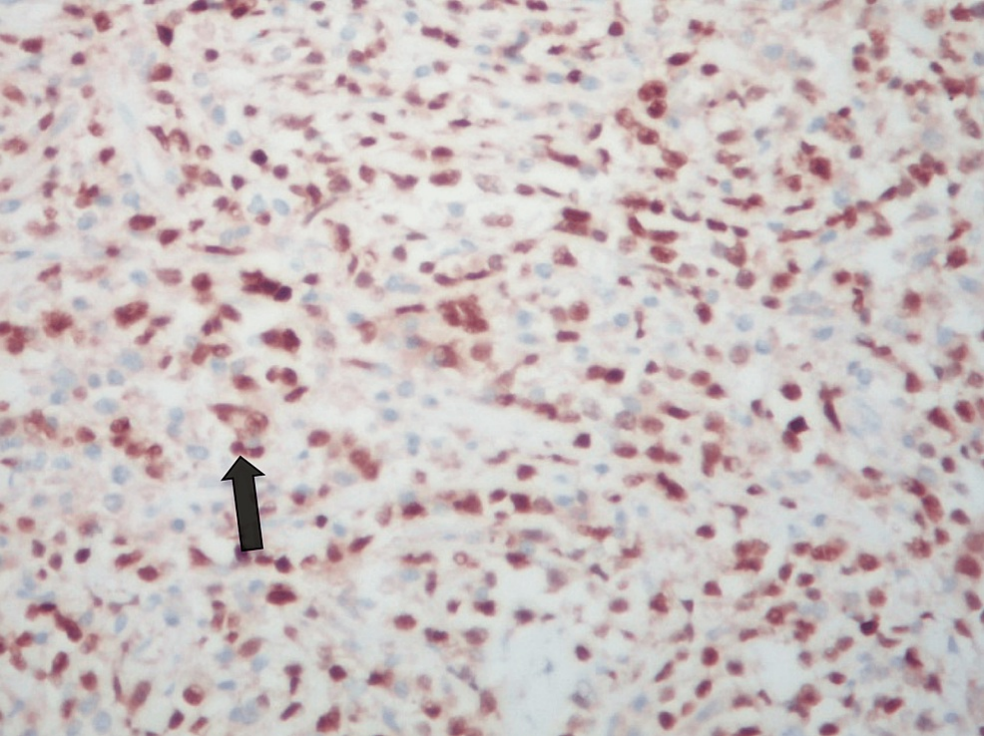
IHC ×400 shows OCT-2 expression.

**Figure 9:**
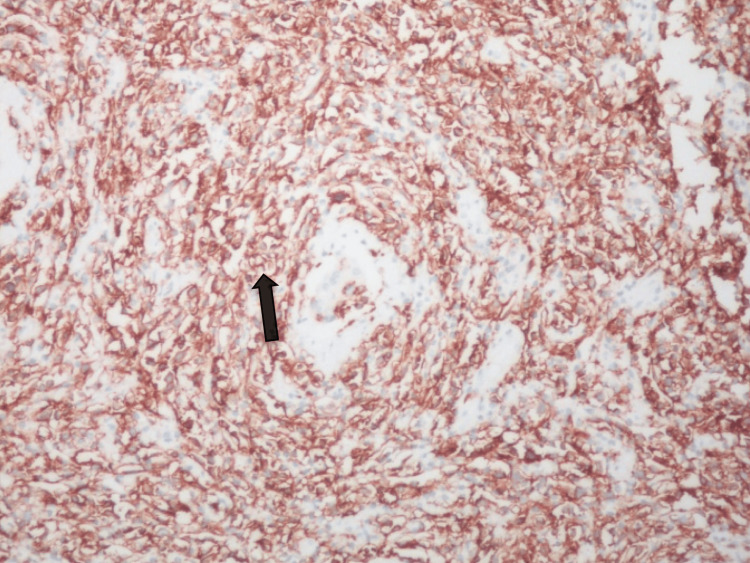
IHC ×200 shows diffuse and strong expression of CD20 in the lymphoid cells.

CD20, CD79a, BCL6, MUM1, PAX5, and OCT2 were expressed in the larger lymphoid cells, which failed to express BCL2, CD30, Fascin, EMA, and PLAP, the latter two being markers of germ cell tumors (Figures 6-9).

**Figure 10:**
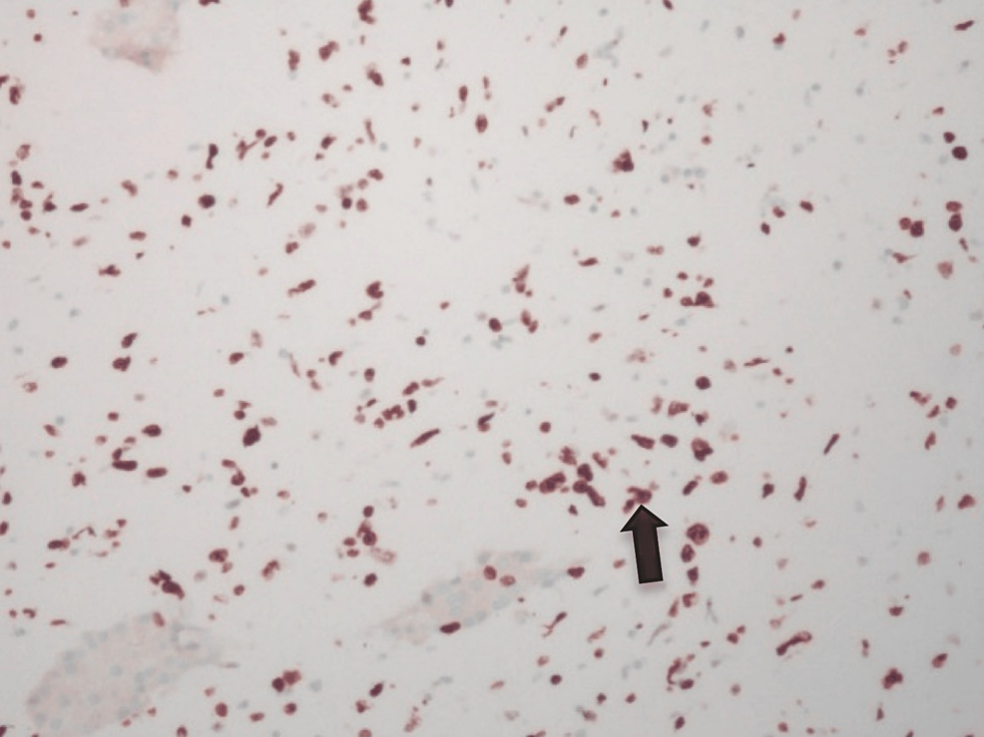
IHC ×200 shows high Ki-67 labeling index (80%) 23-2401.

CD21 failed to identify any FDC meshwork, while EBV was negative. CD10 appeared equivocal. The Ki67 labeling index was >80% (Figure 10).

The findings were in favor of malignant B-cell non-Hodgkin lymphoma, specifically diffuse large B-cell lymphoma (DLBCL).

Post-orchiectomy, the patient was referred to follow up with the oncology team for chemotherapy. There, the patient underwent reevaluation and was staged as stage 4 of testicular DLBCL as testicular and prostatic lymph nodes were involved. The patient had 2 points in the International Prognostic Index for DLBCL (CNS IPI) and 2.4% CNS risk. A lumbar puncture and bone marrow biopsy were done and were unremarkable. The patient then underwent six cycles of Rituximab, cyclophosphamide, doxorubicin, vincristine, and prednisolone (RCHOP), accompanied by two cycles of IT methotrexate and testicular irradiation to prevent recurrence to the other testicle and/or relapse in the CNS. A brain MRI was done, which turned out to be normal with no lymphoma involvement. The end-of-treatment PET scan showed a complete metabolic response.

## Discussion

The rarity of this phenomenon and the unexpected age group of the patient encountered prompted the writing of the present case report, especially considering that NHL is categorized as belonging to the histology group that shows a high incidence rate of bilateral testicular invasion and metastasis.

The mean age of primary testicular NHL at presentation is 60 years, yet there are more studies showing younger ages coinciding with our case [3,8]. However, one study showed a correspondence between age and prognosis, with younger people having a better prognosis [6]. The clinical presentation is usually mistaken for a germ cell tumor if a patient presents with a painless testicular mass, as seen in our case. Seminoma and embryonal carcinoma can be misinterpreted as primary testicular lymphoma and can be distinguished via histology and immunohistochemistry [9]. The patient did not exhibit constitutional symptoms such as fever, weight loss, loss of appetite, fatigue, and night sweats. Frequently, it presents as a unilateral mass, and it can also have bilateral involvement [9,10]. In previous studies, lymphoma was suggested to easily infiltrate the epididymis and other adjacent structures, such as lymph nodes [4,7].

Tumor markers' role in primary testicular NHL correlates with tumor aggressiveness [1,3]. In our case, the βHCG was the only serologic marker of solid tumors that was elevated; others were within the normal range. Ultrasound is an imaging modality used initially; mostly, they are hypoechoic lesions of the testis, either focal or diffuse [11]. In this case, the ultrasound results showed the same picture.

Grossly, the tumor is solid gray-white with a lobulated appearance, replacing testicular tissue [4,7,9]. The pathology in this case was consistent with the other studies. Microscopic features favored the B-cell non-Hodgkin lymphoma subtype as DLBCL, which is consistent with the previous reports [3,6-9,12]. In coincidence with a previous study, our case indicated CD20, which is strongly positive and indicates tumor cell presence [2,7].

Primary testicular NHL treatment includes orchidectomy followed by six cycles of R-CHOP [3,7,8,12,13]. Accompanied by intrathecal (IT) chemotherapy or high-dose systemic methotrexate to prevent reoccurrence in the other testicle and/or relapse in the CNS. High-dose chemotherapy with autologous hematopoietic cell transplantation (HCT) has emerged as an appropriate consolidative therapy after induction treatment in eligible patients (selection of the induction treatment differs based on disease distribution) [14-23]. All patients, except those who are frail, should be evaluated for transplantation eligibility soon after the diagnosis of refractory DLBCL (that did not respond adequately to initial therapy), relapsed DLBCL (that recurs after a complete response was documented), or a secondary CNS DLBCL metastasis [24,25]. The prognosis is often poor, but previous studies showed some of the determining factors for a poor prognosis, such as older age, the presence of systemic symptoms, spermatic cord involvement and bilaterality, vascular invasion, and the level of LDH [3,12]. Common sites of relapse are the central nervous system, which carries a poor prognosis [3,6,9,12]. There is a 50% chance of relapse in the contralateral testis [1,26].

## Conclusions

Although primary testicular lymphoma is most commonly seen in men over the age of 60 years, it has also previously been documented, on occasion, in middle-aged or younger individuals, such as here. The mainstay for diagnosis includes physical examination and tumor markers, with special emphasis on germ cell tumors, followed by US, CT, and histological examination of the excised testicle. Regardless of the stage of lymphoma, orchiectomy is typically insufficient as the only treatment, and the patient will need further treatment such as chemotherapy.
